# Neuroactivity of detonation nanodiamonds: dose-dependent changes in transporter-mediated uptake and ambient level of excitatory/inhibitory neurotransmitters in brain nerve terminals

**DOI:** 10.1186/s12951-016-0176-y

**Published:** 2016-03-31

**Authors:** Natalia Pozdnyakova, Artem Pastukhov, Marina Dudarenko, Maxim Galkin, Arsenii Borysov, Tatiana Borisova

**Affiliations:** Department of Neurochemistry, Palladin Institute of Biochemistry, National Academy of Sciences of Ukraine, 9 Leontovicha Street, Kiev, 01601 Ukraine

**Keywords:** Nanodiamonds, Glutamate, γ-aminobutyric acid, Na^+^-dependent uptake, The extracellular level, Exocytosis, Brain nerve terminals

## Abstract

**Background:**

Nanodiamonds are one of the most perspective nano-sized particles with superb physical and chemical properties, which are mainly composed of carbon sp^3^ structures in the core with sp^2^ and disorder/defect carbons on the surface. The research team recently demonstrated neuromodulatory properties of carbon nanodots with other than nanodiamonds hybridization types, i.e., sp^2^ hybridized graphene islands and diamond-like sp^3^ hybridized elements.

**Results:**

In this study, neuroactive properties of uncoated nanodiamonds produced by detonation synthesis were assessed basing on their effects on transporter-mediated uptake and the ambient level of excitatory and inhibitory neurotransmitters, glutamate and γ-aminobutyric acid (GABA), in isolated rat brain nerve terminals. It was shown that nanodiamonds in a dose-dependent manner attenuated the initial velocity of Na^+^-dependent transporter-mediated uptake and accumulation of l-[^14^C]glutamate and [^3^H]GABA by nerve terminals and increased the ambient level of these neurotransmitters. Also, nanodiamonds caused a weak reduction in acidification of synaptic vesicles and depolarization of the plasma membrane of nerve terminals.

**Conclusions:**

Therefore, despite different types of hybridization in nanodiamonds and carbon dots, they exhibit very similar effects on glutamate and GABA transport in nerve terminals and this common feature of both nanoparticles is presumably associated with their nanoscale size. Observed neuroactive properties of pure nanodiamonds can be used in neurotheranostics for simultaneous labeling/visualization of nerve terminals and modulation of key processes of glutamate- and GABAergic neurotransmission. In comparison with carbon dots, wider medical application involving hypo/hyperthermia, external magnetic fields, and radiolabel techniques can be perspective for nanodiamonds.

## Background

Nanoparticles have great biotechnological potential and wide perspectives for new applications. It is so because properties of nanomaterials often differ from those in bulk forms, and they possess unexpected physical and chemical features. Regarding the central nervous system, investigation of interaction of nanoparticles with neurons showed both negative and positive effects [1, 2]. Nanoparticles can kill the cells by three main pathways, that is, reactive oxygen species formation, mechanical damage of intracellular organelles, and an increase in the cytosolic Ca^2+^ concentration [3]. Therefore, understanding of detailed mechanisms of nanoparticle interaction with the nerve cells is of critical importance for development of new technologies.

Carbon materials attract a lot of attention regarding their new unusual properties that can be implemented in nanotechnology. Among carbons, nanodiamonds (NDs) are one of the most perspective nano-sized particles due to their unique physical and chemical properties, e.g., excellent mechanical and optical properties, high surface areas and tunable surface structures [4–6]. They are mainly composed of carbon sp^3^ structures in the core, with sp^2^ and disorder/defect carbons on the surface [6]. NDs are produced predominately by two methods, that is, using high temperature/high pressure or detonation, and also laser ablation and plasma-enhanced chemical vapor deposition can be employed to produce NDs for different applications [4–9]. Owing to its surface properties, which allow modification and conjugation of a variety of biofunctional entities for controlled targeted drug delivery, in particular water-insoluble drugs, and better penetration of the drug complex inside cells, NDs is a perspective material among others with a wide range of potential applications in tribology, drug delivery, bioimaging and tissue engineering, and also as a filler material for nanocomposites [4, 6, 10]. NDs were perspective as a drug delivery system for treatment of malignant brain gliomas [11]. Also, they have unique thermal properties.

NDs’ physical and chemical properties open possibilities for their use in theranostics. Due to the growth/production procedures, a large number of lattice defects exist in the core of NDs, which form fluorescent color centers. The centers can be excited with almost any excitation wavelength; emitted fluorescence is stable and the photobleaching is limited. Moreover, the defect centers can be enhanced with high-energy beam treatment followed by thermal annealing [12–16].

NDs are considered as non-toxic agents that make them well suited to a wide range of biomedical applications [4]. Many existing NDs-related results focus on the cellular models or micro-organisms, and so there is a need to carry out research at the more complicated levels, e.g., using animal models, for further progress in these studies. The interaction of NDs with animal organs and tissues, circulation in the organism, and NDs clearance in the animal body has not been systematically studied [6]. Despite the potential of NDs in drug delivery has been demonstrated, fundamental mechanisms of their interaction with the cells are still poorly understood.

Recently, the authors of the present study revealed significant neuroactive properties of carbon nanodots (CDs), nanoparticles with other than NDs type of hybridization. CDs are characterized by non-fluorescent carbon core with sp^2^ hybridized graphene islands and diamond-like sp^3^ hybridized elements. Fluorescent carbon dots obtained from β-alanine by microwave heating significantly influenced presynaptic transport of glutamate and γ-aminobutyric acid (GABA), which are key excitatory and inhibitory neurotransmitters in the mammalian central nervous system [17]. The ambient level of glutamate and GABA between the episodes of exocytotic release is maintained at a definite range by permanent transporter-mediated turnover of the neurotransmitters across the plasma membrane [18, 19]. Na^+^-coupled neurotransmitter transporters play a key role in the termination of synaptic neurotransmission and mediate uptake of amino acid neurotransmitters into the cytosol. The transporters are plasma membrane proteins with several transmembrane domains, and they use Na^+^/K^+^ electrochemical gradients as a driving force for transfer of the neurotransmitters across the plasma membrane [20–22].

The present study focused on the estimation of the effects of pure uncoated NDs produced by detonation synthesis on the key characteristics of glutamatergic and GABAergic neurotransmission in isolated rat brain nerve terminals, thereby uncovering their possible neuroactive properties.

## Methods

### Synthesis of NDs

NDs for our experiments were obtained according to Orel et al. [9] by the method of detonating synthesis using a detonation wave at the explosion of powerful explosive material with negative oxygen balance (trotyl/hexogen, grade TG-40/60). Specific magnetic susceptibility of the preparation consisted of 154.7 × 10^−8^ m^3^/kg. Unburned residue in the preparation, e.g., metals and ceramics, was equal to 4.7 % [9].

### Ethical approval

Wistar male rats, 100–120 g body weight, were obtained from the vivarium of M.D. Strazhesko Institute of Cardiology, Medical Academy of Sciences of Ukraine. Animals were kept in animal facilities of the Palladin Institute of Biochemistry, housed in a quiet, temperature-controlled room (22–23 °C) and were provided with water and dry food pellets ad libitum. All procedures were conducted according to the Declaration of Helsinki (“Scientific Requirements and Research Protocols” and “Research Ethics Committees”). Experimental protocols were approved by the Animal Care and Use Committee of the Palladin Institute of Biochemistry (Protocol from 19/09-2011). Before removing the brain, rats were sacrificed by rapid decapitation. The total number of animals used in the study was 16, i.e., the assessment of glutamate uptake—4 animals and the extracellular level—4 animals (4 animals per parameter); GABA uptake—4 animals and the extracellular level—4 animals (4 animals per parameter).

### Isolation of rat brain nerve terminals (synaptosomes)

Cerebral hemispheres of decapitated animals were rapidly removed and homogenized in ice-cold 0.32 M sucrose, 5 mM HEPES–NaOH, pH 7.4, and 0.2 mM EDTA. One animal was used to obtain one synaptosomal preparation, and each measurement was performed in triplicate. The synaptosomes were prepared by differential and Ficoll-400 density gradient centrifugation of rat brain homogenate according to the method of Cotman [23] with slight modifications [24]. All manipulations were performed at 4 °C. The synaptosomal suspensions were used in experiments during 2–4 h after isolation. The standard saline solution was oxygenated and contained (in mM): NaCl 126; KCl 5; MgCl_2_ 2.0; NaH_2_PO_4_ 1.0; CaCl_2_ 2; HEPES 20, pH 7.4; and d-glucose 10. Protein concentration was measured as described by Larson et al. [25].

### l-[^14^C]glutamate uptake by nerve terminals

Uptake of l-[^14^C]glutamate by synaptosomes was measured as follows. Synaptosomal suspension (125 μl; of the suspension, 0.2 mg of protein/ml) was pre-incubated in standard saline solution at 37 °C for 10 min, then NDs (0.05–1 mg/ml) were added to the synaptosomal suspension and incubated for 5 min. Uptake was initiated by the addition of 10 µM l-glutamate supplemented with 420 nM l-[^14^C]glutamate (0.1 μCi/ml), incubated at 37 °C during different time intervals (1, 2, 10 min) and then rapidly sedimented using a microcentrifuge (20 s at 10,000 *g*). l-[^14^C]glutamate uptake was determined as a decrease in radioactivity in aliquots of the supernatant (100 μl) and an increase in radioactivity of the pellet (SDS-treated) measured by liquid scintillation counting with ACS scintillation cocktail (1.5 ml) [26]. Data collected in triplicate in four independent experiments performed with different synaptosomal preparations each are presented as mean ± SEM.

### [^3^H]GABA uptake by nerve terminals

Synaptosomes were diluted in standard saline solution containing GABA transaminase inhibitor aminooxyacetic acid (100 μM) to minimize formation of GABA metabolites. Concentration of protein in synaptosomal samples was 200 μg/ml. Samples were preincubated at 37 °C for 10 min, then NDs (0.05–1 mg/ml) were added to the synaptosomal suspension and incubated for 5 min. Uptake was initiated by the addition of GABA and [^3^H]GABA (1 μM and 50 nM–0.1 μCi/ml, respectively). GABA uptake was terminated in different time intervals (1, 3, 5 min) by filtering aliquots through a Whatman GF/C filters. After twice washing with 5 ml ice-cold standard saline, filters were dried, then were suspended in Organic Counting Scintillant and counted in a Delta 300 (Tracor Analytic, USA) scintillation counter. Non-specific binding of the neurotransmitter was evaluated in cooling samples filtrated immediately after the addition of radiolabelled GABA. Data are mean ± SEM of four independent experiments each performed with different synaptosomal preparations in triplicate.

### Measurements of the ambient level of l-[^14^C]glutamate in the preparation of nerve terminals

Synaptosomes were diluted in standard saline solution to reach concentration of 2 mg of protein/ml and after pre-incubation at 37 °C for 10 min they were loaded with l-[^14^C]glutamate (1 nmol/mg of protein, 238 mCi/mmol) in oxygenated standard saline solution at 37 °C for 10 min. After loading, suspension was washed with ten volumes of ice-cold oxygenated standard saline solution; the pellet was re-suspended in a solution to a final concentration of 1 mg protein/ml and immediately used for release experiments. Synaptosomal suspension (125 μl; 0.5 mg of protein/ml) was pre-incubated for 10 min at 37 °C, then the NDs (0.05–1 mg/ml) were added and incubated for 5 min and then rapidly sedimented using a microcentrifuge (20 s at 10,000*g*). Release was measured in the aliquots of the supernatants (100 μl) and pellets by liquid scintillation counting with scintillation cocktail ACS (1.5 ml). The result was expressed in nmol of l-[^14^C]glutamate/mg of protein [27]. Data collected in triplicate in four independent experiments performed with different synaptosomal preparations each are presented as mean ± SEM.

### Measurements of the ambient level of [^3^H]GABA in the preparation of nerve terminals

Synaptosomes were diluted in standard saline solution to 2 mg of protein/ml and after pre-incubation for 10 min at 37 °C were loaded with [^3^H]GABA (50 nM, 4.7 μCi/ml) in the oxygenated standard saline solution for 10 min. 100 μM aminooxyacetic acid was present throughout all experiments of [^3^H]GABA loading and release. After loading, the suspension was washed with ten volumes of ice-cold oxygenated standard saline solution. The pellet was re-suspended in a standard saline solution to obtain protein concentration of 1 mg of protein/ml. Synaptosomal suspension (120 μl) was pre-incubated for 10 min at 37 °C, then the NDs (0.05–1 mg/ml) were added and incubated for 5 min and then rapidly sedimented using a microcentrifuge (20 s at 10,000*g*). [^3^H]GABA was measured in the aliquots of supernatants (90 μl) by liquid scintillation counting with scintillation cocktail ACS (1.5 ml) and expressed in pmol of [^3^H]GABA/mg of protein [28]. Data are mean ± SEM of four independent experiments each performed with different synaptosomal preparations in triplicate.

### Measurement of synaptosomal plasma membrane potential (*E*_m_)

Membrane potential was measured using a potentiometric fluorescent dye rhodamine 6G (0.5 μM) based on its potential-modulated binding to the plasma membrane [29]. The suspension of synaptosomes (0.2 mg/ml of final protein concentration) after preincubation at 37 °C for 10 min was added to stirred thermostated cuvette. To estimate changes in the plasma membrane potential the ratio (*F*) as an index of membrane potential was calculated according to Eq 1:

1 $$F = {{F_{\text{t}} } \mathord{\left/ {\vphantom {{F_{\text{t}} } {F_{0} }}} \right. \kern-0pt} {F_{0} }}$$

where *F*_0_ and *F*_*t*_ are fluorescence intensities of a fluorescent dye in the absence and presence of the synaptosomes, respectively. *F*_0_ was calculated by extrapolation of exponential decay function to *t* = 0.

Fluorescence measurements with Rhodamine 6G were carried using a Hitachi MPF-4 spectrofluorimeter at 528 nm (excitation) and 551 nm (emission) wavelengths (slit bands 5 nm each).

### Measurements of synaptic vesicle acidification in the synaptosomes

Acridine orange, a pH-sensitive fluorescent dye, is known to selectively accumulate by the acid compartments of synaptosomes (synaptic vesicles). Therefore it was used for monitoring synaptic vesicle acidification. Fluorescence changes were measured using a Hitachi MPF-4 spectrofluorimeter at excitation and emission wavelengths of 490 and 530 nm, respectively (slit bands 5 nm each). Reaction was started by the addition of acridine orange (final concentration 5 μM) to synaptosomal suspension (0.2 mg/ml of final protein concentration) preincubated in a stirred thermostated cuvette at 30 °C for 10 min. The equilibrium level of dye fluorescence was achieved after 3 min. Fluorescence (*F*) was determined according to Eq. (1).

### Statistical analysis

Results were expressed as mean ± S.E.M. of *n* independent experiments. The difference between two groups was compared by two-tailed Student’s *t* test. Differences were considered significant when P ≤ 0.05.

### Materials

EDTA, HEPES, aminooxyacetic acid, d-glucose, sucrose, rotenone, oligomycin, Whatman GF/C filters, analytical grade salts were purchased from Sigma (St. Louis, MO, USA); Ficoll 400, l-[^14^C]glutamate, aqueous counting scintillant (ACS), organic counting scintillant (OCS) were from Amersham (Little Chalfont, UK); [^3^H]GABA (γ-[2,3-^3^H(N)]-aminobutyric acid) was from Perkin Elmer (Waltham, MA, USA).

## Results

### Effect of NDs on the functioning of high-affinity Na^+^-dependent neurotransmitter transporters in nerve terminals

The experiments were carried out in the suspension of nerve terminals isolated from rat brain cerebral hemispheres (synaptosomes). They retain all characteristics of intact nerve terminals, that is, the ability to maintain membrane potential, accomplish uptake and transporter-mediated release of glutamate, exocytosis, endocytosis, etc. Synaptosomes are one of the best systems to explore the relationship between the structure of a protein, its biochemical and cell-biological properties, and physiological role [30].

In the experiments, the NDs were added to synaptosomal suspension 5 min before starting high-affinity transporter-mediated uptake process by l-[^14^C]glutamate or [^3^H]GABA, so the acute effects of NDs were analyzed. It should be noted that glutamate and GABA transporters belong to the different families, i.e., glutamate transporters belong to the SLC1 family, whereas GABA transporters (as well as carriers for the biogenic monoamines and glycine) belong to the SLC6 family [31].

Before the experiments with synaptosomes, water suspension of NDs at a concentration of 2 mg/ml was subjected to ultrasound treatment at 22 kHz for 1 min.

#### Effect of NDs on transporter-mediated l-[^14^C]glutamate uptake by nerve terminals

**Fig. 1:**
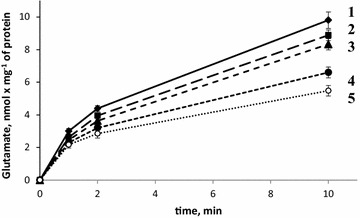
Time course of l-[^14^C]glutamate uptake by synaptosomes in control (*1*) and in the presence of NDs at a concentration of 0.05 mg/ml (*2*); 0.1 mg/ml (*3*); 0.5 mg/ml (*4*) and 1 mg/ml (*5*). Data is mean ± SEM of four independent experiments, each of them was performed with different synaptosomal preparations in triplicate

Influence of NDs on the initial velocity of l-[^14^C]glutamate uptake by synaptosomes was analyzed. As shown in Fig. 1, the addition of NDs to synaptosomes caused significant changes in the initial velocity of l-[^14^C]glutamate uptake that was equal to 3.0 ± 0.17 nmol min^−1^ mg^−1^ protein in the control experiments, and 2.62 ± 0.14 nmol min^−1^ mg^−1^ protein in the presence of NDs at a concentration of 0.05 mg/ml; 2.46 ± 0.18 nmol min^−1^ mg^−1^ protein—NDs at a concentration of 0.1 mg/ml; 2.3 ± 0.16 nmol min^−1^ mg^−1^ protein—0.5 mg/ml of NDs (p < 0.05, Student’s *t* test, n = 4) and 2.17 ± 0.2 nmol min^−1^ mg^−1^ protein – 1 mg/ml of NDs (p < 0.05, Student’s *t* test, n = 4).

Accumulation of l-[^14^C]glutamate by synaptosomes for 10 min consisted of 9.8 ± 0.5 nmol min^−1^ mg^−1^ protein in the control experiments, and 8.88 ± 0.33 nmol min^−1^ mg^−1^ protein in the presence of NDs at a concentration of 0.05 mg/ml; 8.33 ± 0.35 nmol min^−1^ mg^−1^ protein—NDs at a concentration of 0.1 mg/ml; 6.6 ± 0.33 nmol min^−1^ mg^−1^ protein—0.5 mg/ml of NDs (p < 0.05, Student’s *t* test, n = 4) and 5.48 ± 0.32 nmol min^−1^ mg^−1^ protein—1 mg/ml of NDs (p < 0.05, Student’s *t* test, n = 4). Thus, we observed that NDs inhibited l-[^14^C]glutamate uptake and its accumulation by synaptosomes in a dose-dependent manner.

#### Effect of NDs on transporter-mediated [^3^H]GABA uptake by nerve terminals

**Fig. 2:**
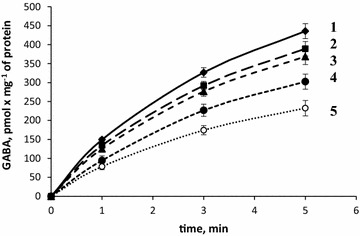
Time course of [^3^H]GABA uptake by synaptosomes in control (*1*) and in the presence of NDs at a concentration of 0.05 mg/ml (*2*); 0.1 mg/ml (*3*); 0.5 mg/ml (*4*) and 1 mg/ml (*5*). Data is mean ± SEM of four independent experiments, each of them was performed with different synaptosomal preparations in triplicate

As shown in Fig. 2, NDs decreased the initial velocity of [^3^H]GABA uptake by synaptosomes that consisted of 149.4 ± 5.5 pmol min^−1^ mg^−1^ protein in the control and 134.4 ± 4.4 pmol min^−1^ mg^−1^ protein in the presence of NDs at a concentration of 0.05 mg/ml; 125.2 ± 8.6 pmol min^−1^ mg^−1^ protein—NDs at a concentration of 0.1 mg/ml; 94.5 ± 12.3 pmol min^−1^ mg^−1^ protein—0.5 mg/ml of NDs (p < 0.05, Student’s *t* test, n = 4) and 78.5 ± 8.4 pmol min^−1^ mg^−1^ protein—1 mg/ml of NDs (p < 0.001, Student’s *t* test, n = 4).

Accumulation of [^3^H]GABA by synaptosomes for 5 min consisted of 435.7 ± 19.8 pmol min^−1^ mg^−1^ protein in control and 232.5 ± 20.3 pmol min^−1^ mg^−1^ protein in the presence of NDs at a concentration of 1 mg/ml (p < 0.001, Student’s *t* test, n = 4). Therefore, similarly with the experiments with l-[^14^C]glutamate, the NDs caused an immediate decrease in the initial velocity of uptake and accumulation of [^3^H]GABA by synaptosomes in a dose-dependent manner.

### Influence of NDs on the ambient level of the neurotransmitters in the preparations of nerve terminals

Definite level of ambient glutamate and GABA, and so proper balance of excitatory/inhibitory signals determines normal synaptic transmission, whereas the changes in this level and misbalance of excitation and inhibition can provoke the development of neurological consequences. The ambient level of the neurotransmitters is determined mainly by permanent neurotransmitter turnover, that is, balance of transporter-mediated uptake/release and non-transporter tonic release in nerve terminals [18]. NDs-induced decrease in transporter-mediated uptake of glutamate and GABA shown in the previous subsection is expected to result in an increase in the extracellular level of theses neurotransmitters in the nerve terminals, similar to that shown by the authors with carbon dots [17], and also using cholesterol-deficiency models [32], however, this correlation was not confirmed by specific centrifuge-induced hypoxia model [33, 34].

#### Effects of NDs on the ambient level of l-[^14^C]glutamate in the preparations of nerve terminals

**Fig. 3:**
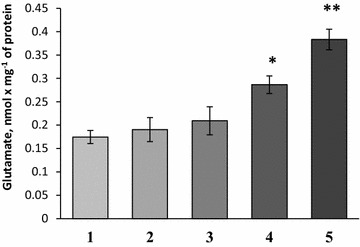
The extracellular level of l-[^14^C]glutamate in synaptosomal suspension in control (*1*) and in the presence of NDs at a concentration of 0.05 mg/ml (*2*); 0.1 mg/ml (*3*); 0.5 mg/ml (*4*) and 1 mg/ml (*5*). Data is mean ± SEM of four independent experiments, each of them was performed with different synaptosomal preparations in triplicate. *p < 0.05; **p < 0.001 as compared to control

As shown in Fig. 3, NDs caused significant changes in the extracellular level of l-[^14^C]glutamate in synaptosomal suspension. The extracellular level of l-[^14^C]glutamate in synaptosomal preparations consisted of 0.174 ± 0.014 nmol mg^−1^ of protein in the control (column #1) and 0.19 ± 0.026 nmol mg^−1^ of protein in the presence of NDs at a concentration of 0.05 mg/ml (column #2); 0.209 ± 0.03 nmol mg^−1^ of protein—0.1 mg/ml of NDs (column #3); 0.286 ± 0.019 nmol mg^−1^ of protein—0.5 mg/ml of NDs (p < 0.05, Student’s *t* test, n = 4) (column #4) and 0.383 ± 0.022 nmol mg^−1^ of protein—1 mg/ml of NDs (p < 0.001, Student’s *t* test, n = 4) (column #5). Therefore, NDs considerably increased the ambient level of l-[^14^C]glutamate in synaptosomes.

#### Effects of NDs on the ambient level of [^3^H]GABA in the preparations of nerve terminals

**Fig. 4:**
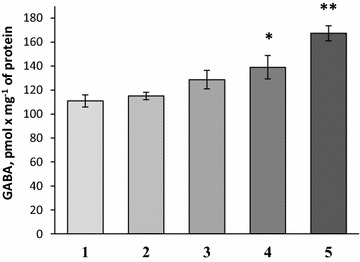
The extracellular level of [^3^H]GABA in synaptosomes in control (*1*) and in the presence of NDs at a concentration of 0.05 mg/ml (*2*); 0.1 mg/ml (*3*); 0.5 mg/ml (*4*) and 1 mg/ml (*5*). Data is mean ± SEM of four independent experiments, each of them was performed with different synaptosomal preparations in triplicate. *p < 0.05; **p < 0.001 as compared to control

The extracellular level of [^3^H]GABA was analyzed in synaptosomal suspension in the presence of NDs at different concentrations. After 5 min of incubation of synaptosomes with NDs (Fig. 4), the extracellular level of [^3^H]GABA in synaptosomal suspension was equaled to 110.9 ± 5.11 pmol mg^−1^ of protein in the control (column #1) and 115.05 ± 3.04 pmol mg^−1^ of protein in the presence of NDs at a concentration of 0.05 mg/ml (column #2); 128.64 ± 7.73 pmol mg^−1^ of protein—0.1 mg/ml of NDs (column #3); 138.96 ± 9.73 pmol mg^−1^ of protein—0.5 mg/ml of NDs (p < 0.05, Student’s *t* test, n = 4) (column #4) and 167.37 ± 6.24 pmol mg^−1^ of protein—1 mg/ml of NDs (p < 0.001, Student’s *t* test, n = 4) (column #5). Therefore, a significant increase in the extracellular level of [^3^H]GABA in synaptosomes in the presence of NDs was found (similarly with the experiments with l-[^14^C]glutamate).

### Membrane potential of nerve terminals in the presence of NDs

The key parameters that can significantly alter the functioning of Na^+^-dependent transporters of neurotransmitters and their extracellular level are: (1) the potential of the plasma membrane of nerve terminals, because Na^+^/K^+^ electrochemical gradient across the plasma membrane serves as a driving force for glutamate and GABA transporter functioning; and (2) acidification of synaptic vesicles. The potential was measured using the cationic potentiometric dye rhodamine 6G, which binds to the negative charges of the membranes (see “Methods” section).

**Fig. 5:**
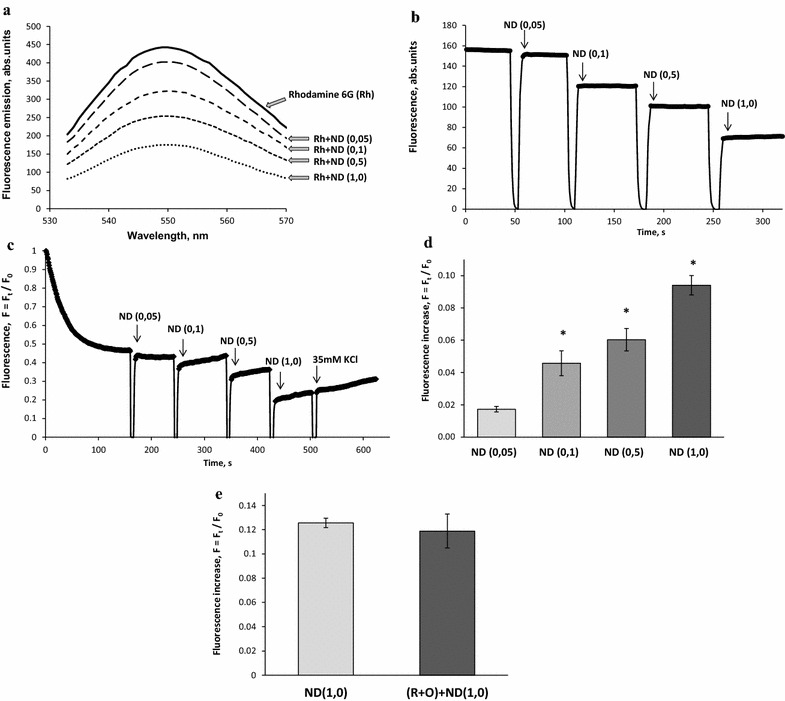
**a** Fluorescence emission spectra of rhodamine 6G (0.5 μM) in the standard salt solution before and after application of NDs (0.05–1.0 mg/ml). **b** Quenching of fluorescence signal of rhodamine 6G (0.5 μM) in the presence of NDs (0.05–1.0 mg/ml) without synaptosomes; **c** Dose-dependent effect of NDs (0.05–1.0 mg/ml) on the membrane potential of synaptosomes; **d** an increase in the fluorescence signal of rhodamine 6G in response to application of NDs (0.05–1.0 mg/ml); **e** an increase in the fluorescence signal of rhodamine 6G in response to application of NDs (1.0 mg/ml) without (*the first column*) and in the presence of 5 µM rotenone and 5 µg/ml oligomycin (R + O) (*the second column*). The suspension of synaptosomes was equilibrated with potential-sensitive dye rhodamine 6G (0.5 µM); when the steady level of the dye fluorescence had been reached, NDs at concentrations 0.05–1.0 mg/ml were added (marked by *arrows*) to synaptosomes. Trace represents four experiments performed with different preparations. Data is mean ± SEM. *p < 0.05 as compared to the steady level of the dye fluorescence

In the next series of the experiments, it was assessed whether or not NDs influenced the fluorescence of rhodamine 6G. No significant changes were found in the emission spectrum of rhodamine 6G in response to the addition of NDs at concentrations within the range 0.05–1.0 mg/ml (Fig. 5a). In the experiments without synaptosomes, the fluorescence signal of rhodamine 6G was quenched in response to addition of NDs to its solution in cuvette (Fig. 5b).

As shown in Fig. 5c, the addition of synaptosomal suspension to the medium containing rhodamine 6G was accompanied by a partial decrease in fluorescence due to binding of the dye to the plasma membrane. F_st_, the membrane potential index at the steady state level, was achieved after 3 min. It was demonstrated that NDs at a concentration of 0.05 mg/ml did not influence significantly the fluorescence signal of rhodamine 6G (Fig. 5c, d). The changes in the membrane potential, and so membrane depolarization can be registered starting from concentrations of NDs in the incubation media equal to 0.1 mg/ml, whereas the application of 0.5 and 1.0 mg/ml of NDs led to a significant depolarization of the plasma membrane of nerve terminals. However, NDs did not affect completely the ability of synaptosomes to be depolarized in respond to the addition of high-KCl (Fig. 5c).

Rhodamine 6G was bounded to both plasma and mitochondrial membranes in accordance to their potentials. The contribution of the plasma membrane potential to the dye fluorescence was assessed under conditions of collapsed mitochondrial potential by treatment with rotenone (5 μM) and oligomycin (5 μg/ml), the specific inhibitors of the mitochondrial respiratory chain and ATP synthase, respectively. The comparative analysis revealed the similarity in NDs-evoked increase in rhodamine 6G fluorescence in norm and under conditions of collapsed mitochondrial potential (Fig. 5e).

### Effects of NDs on acidification of synaptic vesicles

The question rose whether or not NDs-mediated impairment of l-[^14^C]glutamate and [^3^H]GABA uptake by synaptosomes (shown in the previous subsection) resulted from the changes in synaptic vesicle acidification. To answer this query, a pH-sensitive fluorescent dye acridine orange was used in order to measure the synaptic vesicle acidification, which is an important component of electrochemical proton gradient. This suggestion follows from our recent results demonstrating that the treatment of synaptosomes with 200 nM bafilomycin A1 (a specific inhibitor of the vacuolar type H^+^-ATPase) for 20 min lowered the initial velocity of l-[^14^C]glutamate uptake from 2.5 ± 0.3 to 1.1 ± 0.2 nmol min^−1^ mg^−1^ protein. So, the inhibition of vesicular uptake by bafilomycin A1 caused a significant decrease in the activity of synaptosomal l-[^14^C]glutamate uptake.

**Fig. 6:**
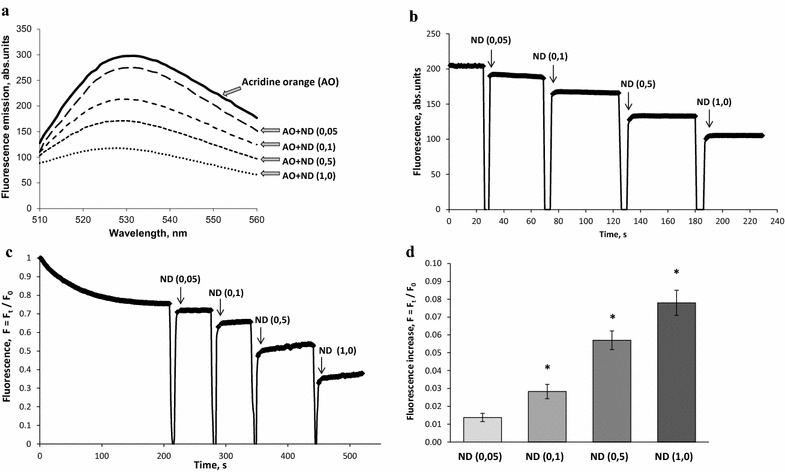
**a** Fluorescence emission spectra of acridine orange (5 µM) in the standard salt solution before and after application of NDs (0.05–1.0 mg/ml). **b** Quenching of fluorescence signal of acridine orange in the presence of NDs (0.05–1.0 mg/ml) without synaptosomes; **c** acidification of synaptosomes in the presence of NDs (0.05–1.0 mg/ml); **d** an increase in the fluorescence signal of acridine orange in response to application of NDs (0.05–1.0 mg/ml). The synaptosomes were equilibrated with acridine orange (5 µM); when the steady level of the dye fluorescence had been reached, NDs at concentrations 0.05–1.0 mg/ml (*arrows*) were added. Trace represents four experiments performed with different preparations. Data is mean ± SEM. *p < 0.05 as compared to the steady level of the dye fluorescence

At first, it was assessed whether or not NDs influenced acridine orange fluorescence. No significant changes were found in the emission spectrum of acridine orange in response to the addition of NDs within the concentration range 0.05–1.0 mg/ml (Fig. 6a). It was also demonstrated that without synaptosomes NDs caused a quenching of fluorescence signal of acridine orange (Fig. 6b). As shown in Fig. 6c, the application of acridine orange to synaptosomes resulted in partial quenching of fluorescence signal due to dye accumulation in synaptic vesicles. After loading of synaptosomes with acridine orange, NDs were added to the incubation medium that resulted in an increase in the fluorescence of acridine orange indicating a decrease in acidification of synaptic vesicles (Fig. 6c, d). These changes can be registered starting from concentrations of NDs in the incubation media equal to 0.1 mg/ml. Therefore, the causes that led to a decrease in transporter-mediated l-[^14^C]glutamate and [^3^H]GABA uptake by synaptosomes in the presence of NDs could be the depolarization of their plasma membrane and dissipation of the proton gradient of synaptic vesicles.

## Discussion

In the past few years, biocompatibility and toxicity of NDs has been intensively investigated and a progress concerning their biological and medical application for imaging and therapy has been achieved [6, 9, 35]. Literature data revealed that pure/surface functionalized and conjugated NDs interacted with different types of cell cultures and tissues [6, 36–40]. Liu et al. [41] investigated the location and distribution of 100 nm carboxylated NDs during cell division and differentiation. The NDs were entering into the cells by macropinocytosis and clathrin-mediated endocytosis pathways, and the growth ability of the cells was not altered by endocytic NDs after long-term cell culture for 10 days in both A549 lung cancer cells and 3T3-L1 embryonic fibroblasts. In the presence of NDs, the daughter cells after cell division not only survived but also continued to divide further. The NDs treatment did not affect the gene or protein expression, or the regulation of cell cycle progression at adipogenic differentiation. In addition, the NDs did not alter the cell cycle-regulated protein levels, total cell number or percentage of cell cycle phases in both A549 and 3T3-L1 cells in long-term culture. So, the findings proved that endocytic NDs were noncytotoxic for cell division and differentiation [6, 42]. Therefore, a low toxicity of NDs (with the size of appx. 100 nm) was shown for different cell functions (including division, gene expression and immune response) suggesting biocompatibility of NDs in the cellular level [6]. However, the fundamental mechanisms of NDs action on the cells are still poorly understood.

Neuroactivity of NDs was assessed at the neurochemical level of the central nervous system organization. Neurochemical parameters, that is, uptake and the ambient level of glutamate and GABA in nerve terminals was analyzed according to *Guidelines for Neurotoxicity Risk Assessment* of US Environmental Protection Agency, 1998, based on paragraph 3. *Hazard Characterization*: 3.1.2. *Animal Studies*; 3.1.2.3.*Neurochemical Endpoints of Neurotoxicity*; 3.1.3.4.*In Vitro Data in Neurotoxicology*.

In this study, neuroactive effects of NDs were shown using nerve terminals. The NDs within the concentration range from 0.05 to 1 mg/ml attenuated the initial velocity of transporter-mediated uptake of l-[^14^C]glutamate (Fig. 1) and [^3^H]GABA (Fig. 2) in a dose-dependent manner, and increased the ambient level of l-[^14^C]glutamate (Fig. 3) and [^3^H]GABA (Fig. 4) in the preparation of nerve terminals. Parameters analyzed in this study, that is, uptake and ambient level of the neurotransmitters are in tight relation with each other [18, 19], consequently, NDs-evoked attenuation of the initial velocity of neurotransmitter uptake is associated with their weak transport to the cytosol of nerve terminals, and so the enhancement of the ambient level. NDs-induced changes in above parameters of glutamate and GABA transport can change balance between excitation and inhibition processes. It is so because the maintenance of definite ambient concentration of glutamate and GABA between the episodes of exocytotic release is particularly important for tonic activation of excitatory and inhibitory post- and pre-synaptic receptors of these neurotransmitters. Also, during exocytotic events excess of glutamate and GABA in the synaptic cleft (because of weak neurotransmitter uptake) can be accessible for appropriate receptors for prolonged time intervals. It is well known that glial uptake significantly contributes to maintenance of appropriate ambient glutamate concentration in the synaptic cleft. It is expected that NDs are able to affect uptake, the ambient level of l-[^14^C]glutamate and the plasma membrane potential not only in nerve terminals, but also in glial cells.

Taking into account experimental data, it has been suggested that NDs possess neuroactive properties at the concentrations starting from 0.1 to 0.5 mg/ml. From one side, new effects of NDs at above concentrations can be used in appropriate way and technologies (for example, cancer treatment). From the other side, NDs at the concentrations below those exhibited neuroactive effects can be considered non-neuroactive. In perspective, the reversibility of NDs-related effects on uptake and the extracellular level of glutamate and GABA in nerve terminals ought to be analyzed. This data underlies long-term prognosis for development of neurotoxic consequences.

Comparing NDs-related results with recent data of the authors concerning the properties of CDs synthesized from β-alanine, it can be concluded that despite of different type of hybridization in these nanoparticles, their principal neuromodulatory effects were almost similar. Whereas, the strength of their effects was different. CDs in dose-dependent manner decreased exocytotic release of glutamate and GABA, reduced acidification of synaptic vesicles, attenuated the initial velocity of Na^+^-dependent transporter-mediated uptake of glutamate and GABA, increased the ambient level of the neurotransmitters, but nevertheless did not change significantly the potential of the plasma membrane of nerve terminals [17]. CDs synthesized from β-alanine induced much more significant changes in uptake and the extracellular level of l-[^14^C]glutamate and [^3^H]GABA in nerve terminals in comparison with NDs in similar concentrations. Very important characteristic of NDs is their ability to depolarize the plasma membrane of nerve terminals at concentrations more than 0.1 mg/ml. In our experiments, this feature does not inherent to CDs synthesized from β-alanine [17].

Basing on the recent data related to the effects of different nanoparticles on the key characteristics of nerve signal transmission [2, 17, 43, 44], it has been suggested that the size of nanoparticles is of critical importance for exhibition of their neuroactive properties. The size of NDs was less than 10 nm, and so because of this nanoscale range they can enter nerve terminals during endocytosis following synaptic vesicle recycling. If synaptic vesicle function is affected by NDs, transporter-mediated uptake of neurotransmitters also exhibits consequent attenuation [32]. Also, it cannot be excluded that NDs are able to interact/bind with the plasma membrane of nerve terminals and change its physical and chemical properties, and thus can directly affect the functioning of neurotransmitter transporters, and again normal vesicle recycling. In this context, our experimental data is in agreement with the results of Perevedentseva et al. [6], where it was shown that the size of NDs was critical for the development of their cytotoxic effects. With the use of NDs of different size, cytotoxicity tests have demonstrated that NDs with a particle size of approximately 100 nm in diameter with a well-defined diamond structure are nontoxic to many kinds of cultured animal cells, while cytotoxicity of the smaller detonated NDs with crystallite sizes of 3–10 nm (with a high tendency to aggregate, as well as with large amounts of surface disordering) are still under discussion [6, 37, 38]. For medical (clinical) applications, the size of NDs is a limiting factor and an aggregation of NDs is a serious problem, especially for sizes smaller than 50 nm.

Conceptually, the nasal application of NDs in order to influence nerve signal transmission is not excluded but needs additional research. It is so because in a mammalian organism, the nano-sized particles can be efficiently uptaken in nasal, tracheobronchial, and alveolar regions and can be transported along sensory axons of the olfactory nerve to the central nervous system [45–51]. Oberdörster et al. [47] showed that intranasally instilled nano-sized particles can target the central nervous system. TiO_2_ nano-sized particles were found in the brain of exposed 6 week-old male mice [52].

New promising area of the application of detonation NDs can be a combination of their neuroactive and recently discovered radioactive [53] properties. Radioactivity of NDs produced by detonation and static syntheses was revealed. NDs irradiated in the core of a commercial-scale reactor demonstrated the resulting radioactivity that was associated with the presence of metal-containing impurities in the initial NDs. The dose rate of γ-radiation achieved for NDs was about 180 μSv/h and the dose rate of combined γ + β-radiation was ∼720 μSv/h [53].

## Conclusions

Summarizing, detonation NDs possess neuroactive properties, that is, they decrease in a dose-dependent manner the initial velocity of transporter-mediated uptake and accumulation of l-[^14^C]glutamate and [^3^H]GABA in nerve terminals, and increase the ambient level of these neurotransmitters. NDs reduce acidification of synaptic vesicles and cause depolarization of the plasma membrane of nerve terminals. Despite different types of structures in their surface, the common feature of NDs and CDs (synthesized from β-alanine) is the ability to affect glutamate and GABA transport in nerve terminals (however, with different effectiveness), and the proton gradient of synaptic vesicles. These neuromodulatory features of pure uncoated NDs can be practical for labeling and visualization of key processes in nerve terminals, and also in theranostics. NDs have wider potential for medical application in comparison with CDs involving hypo/hyperthermia approach, manipulation by external magnetic fields and radiolabeling.
